# Quotation Accuracy of Systematic Review and Meta-Analysis Protocols on Acupuncture

**DOI:** 10.3390/healthcare10010055

**Published:** 2021-12-29

**Authors:** José M. Morán, María Romero-Moreno, Azucena Santillán-García, Ivan Herrera-Peco

**Affiliations:** 1 Metabolic Bone Diseases Research Group, Nursing Department, Nursing and Occupational Therapy College, Universidad de Extremadura, 10003 Cáceres, Spain; maria_romero_moreno@hotmail.com; 2 University Hospital of Burgos, Universitario de Burgos, 09006 Burgos, Spain; ebevidencia@gmail.com; 3 Nursing Department, Faculty of Medicine, Universidad Alfonso X El Sabio, 28691 Madrid, Spain; iherrpec@uax.es; 4 Pharmacy Department, Faculty of Health Sciences, Universidad Alfonso X El Sabio, 28691 Madrid, Spain

**Keywords:** systematic reviews, systematic review protocols, acupuncture

## Abstract

Currently, published systematic review protocols (SR protocols) have increasingly become a new trend in fields such as acupuncture and are therefore a new source of quotations in these fields. Systematic reviews are considered the pinnacle of the evidence pyramid as they embody comprehensive literature searching. Quotations are key elements to achieve this goal as they can support the assertions of the original authors, but the ‘misquotation’ exists, too, and they can be misleading to the reader. The aim of this study was to examine the quotation accuracy of SR protocols in a meta-analysis on acupuncture research. We searched SCOPUS through 31 December, 2020, and each protocol and its citations were analyzed and classified as correct or incorrect. We used descriptive statistics to report the quotation errors and characteristics of the included protocols. The results showed 248 SR protocols, where 124 protocols received quotations and 38 quotations (31.4%) were erroneous. Only 11 (4.4%) of the published SRs and SR protocols had been published previously. Furthermore, the scientific journal in which the most SR protocols were published was Medicine (193; 77.8%), followed by BMJ Open (39; 15.7%). Authors from China (86.5%) were the most productive in publishing SRs and SR protocols. Finally, we concluded that the number of SR protocols and meta-analyses published in scientific journals and indexed by databases exceeds the publication capacity of the SRs associated with them, generating scientific literature that does not make any novel contribution to knowledge.

## 1. Introduction

The description of the research reported in any field is a cornerstone of the robustness and credibility of its published literature [1]. Therefore, quotations are an inherent and key element of scientific literature. Most often, they confirm authors’ assertions or point to research that is relevant to the reader’s knowledge of a manuscript, therefore allowing them to fully contextualize the manuscript. Quotations are absolutely essential in the reporting of research results in health sciences to explain the rationale or conclusions of research, or, in the case of a review or meta-analysis, for the argumentation of the review or meta-analysis [2]. Previous studies have explored through different approaches the extent to which citations in the scientific literature in health sciences support the assertions of the original authors or whether they are inaccurate [3]. In these studies, quote accuracy involves subjectively verifying whether the results of other studies or the statements of other authors are accurately reflected in the articles citing these “quotes” [4]. Earlier studies in the 1980s reported that the original author was misquoted in up to 15% of cited studies conducted in health sciences and that such errors were so substantial that readers were misled by them. Misquotations are unpleasant for the original author, misleading to the reader, and imply that falsehoods and inaccuracies become “established knowledge” that pollutes the body of knowledge on the topic under study [5]. Quotation error rates vary across journals and disciplines, including 13.7% in burns journals [6], 15% in ophthalmic literature [7], 38% in pediatric orthopedic literature [8], 7.8% [9] and 24% [10] in surgical journals, 11.1% in otolaryngology/head and neck surgery journals [11], 35% in dermatologic literature [12], 12.3% in manual therapy journals [13], 35.2% in emergency medicine literature [14], 20% in biomedical journals [3], and up to 7% in nursing journals [15]. Currently, inaccurate quotations are still common in the biomedical scientific literature, with inappropriate quoting rates of approximately 15%; therefore, the endorsed article was not in line with what was stated by the citing authors [16]. While there is much research on the accuracy of incorporating quotations in studies within different disciplines, there is a large gap in the literature on how accurate the quotation is regarding so-called complementary and alternative medicine (CAM) [17], where acupuncture is one of the leading representatives.

Furthermore, there is no previous research on how the quoting of scientific information within the different protocols of systematic reviews (SR) and meta-analyses is conducted generally and this affects all fields of knowledge. Protocol-based prospective registration of systematic reviews and meta-analyses provides transparency, reduces the potential for bias, and serves to avoid the unintended duplication of reviews. Protocol registration delivers benefits to many involved stakeholders in return for relatively small supplementary effort by researchers registering their systematic and meta-analytic reviews [18]. Protocol publication is possible through various scientific journals that allow such publications or through different organizations, such as Cochrane [19], the Joanna Briggs Institute (JBI) [20], and Campbell Collaboration [21], and even is free of charge through the international prospective registry for systematic reviews and meta-analyses (PROSPERO) [22].

Publishing the SR protocol and meta-analysis is a statement of intent on the part of the researchers that does not commit them beyond the ethical level to eventually undertake the publication of the compromised SR. The first systematic review and meta-analysis protocol on record on acupuncture was published in 2010 [23] and, after a slow start, it was in 2014 when spectacular growth began that has continued to increase the number of SR and meta-analysis protocols on acupuncture published year after year up to the present day. The latest data indicate that the final publication rate, i.e., how many of these acupuncture SR protocols end up as full SRs published in the literature, is 13%, with an average time lag of eight months between the protocol being made public and the SR reaching the literature [23].

Since the number of published SR protocols has rapidly increased in recent years, these protocols are becoming increasingly incorporated as references within a variety of studies. We should not forget that by its very nature, the SR protocol itself may only contribute as a source of knowledge to a study through the background (the protocol contains a brief literature review) or the methodology (it may be replicated or resembled by other studies). A protocol does not contain original information, is not a completed study, does not provide original relevant data, and therefore is not a source of novel knowledge per se.

Given that the number of SR protocols on acupuncture is growing steadily and that this proliferation is leading to an increase in the number of citations for these protocols, we aimed to examine the quotation accuracy of SR protocols and meta-analyses on acupuncture research.

## 2. Materials and Methods

We studied the accuracy of all references to SR protocols and meta-analyses published in scientific journals in the SCOPUS database. Quotation errors were defined as secondary references that did not give credence to the original protocol. The classification of citation and citation errors was confirmed by a second researcher (Ivan Herrera-Peco) with 100% reliability. Each quotation was individually assessed for accuracy and judged to be either correct or incorrect. The original SR and meta-analysis protocol quote was considered correct if it referred to either the background included in the protocol or the methodology. Quotations that considered the protocol as concluded studies, made reference to questions not raised in the protocol, made reference to results not included in the protocol, or considered the protocol as a completed clinical trial were considered incorrect.

### 2.1. Search Strategy

We searched SCOPUS from its inception through 31 December, 2020, using the following search algorithm:

(TITLE (protocol) AND TITLE (meta-analysis) OR TITLE-ABS-KEY (“systematic review”) AND TITLE (acupuncture) AND EXCLUDE (PUBYEAR, 2021)).

No additional restrictions were applied to the search. JMM and IH-P independently reviewed the titles and abstracts of the protocols regarding their relationship to acupuncture research. Full texts were read if necessary for the evaluation and uncertain cases were discussed (only two were noticed). Individual and manual searches were performed for each protocol in the reference lists to the protocols provided by SCOPUS. Full texts of all articles citing SR and meta-analysis protocols were individually screened in duplicates to independently assess the accuracy of the protocol quotation.

Currently, in the literature, only a reasonable volume of SR and meta-analysis protocols associated with acupuncture is available and, for this reason, acupuncture was chosen within the CAM category.

### 2.2. Data Collection

Each protocol and its citations were shared among the team members through Google Sheets. We collected information identifying the protocol (doi, journal, and country), the year of publication, the number of citations received, whether the review associated with the protocol had been published, the year of publication of the review, the time elapsed between the publication of the protocol and the review if applicable, the number of correct quotations, the number of incorrect quotations, the references not verifiable due to a lack of access to the quoting article, the incorrect citation (doi), and the author’s e-mail address for correspondence.

To confirm whether the publication of the review associated with the protocol was completed, the articles registered in SCOPUS by each author (first, senior, and correspondence) were reviewed. All authors also were contacted by email regarding those acupuncture research protocols for which a final SR associated with the protocol in question cannot be located. Contact was made to determine whether the publication had been published (and was not registered in any of the journals covered by SCOPUS), whether it was in progress, whether it had not been published, or whether the project had been discontinued. Each corresponding author was contacted via the email address provided in the published protocol and we waited for a response for a total of 30 days. After that time, it was considered a non-response to the request for information.

In previous literature analyzing quotations from completed studies, most authors classified errors into two categories, as they qualitatively assessed the degree of accuracy with which the citing authors referred to the content of the original article [2,5]. Thus, quotation errors were usually assessed as “more serious” or “less serious”. However, this classification is not applicable in our study, since the protocol, by its very nature, does not provide original data; therefore, any reference to its content beyond the information it may provide as background or methodology must be considered erroneous. In this case, the quotations analyzed in this study were classified into two categories: either correct or incorrect.

## 3. Results

A total of 252 SR protocols and meta-analyses on acupuncture research were identified until December 2020. Two records were excluded because they corresponded to errata associated with protocols already included in the database. Two quoted protocols were excluded from the analysis because they did not correspond to SR and meta-analysis protocols (one corresponded to an erratum and the other to a clinical trial protocol). Of the 248 SR protocols included in the study, 124 received quotations. A total of 121 citations to these 124 protocols were reviewed. The most cited had a total of 12 citations and the median (IQR) was one (two) citation. A total of 124 protocols (50%) had no references at all. A total of 38 (31.4%) of the reviewed quotations were erroneous, 109 (90%) were correct, and 18 (14.8%) could not be verified.

When we analyzed the erroneous quotations, we observed that the common error in quotation was the use of the protocol of SR as a real SR or meta-analysis that offered validated scientific evidence used in the manuscripts’ discussion section (37; 97.37%). Additionally, we found that only in one case (1; 2.63%), the protocol of SR was cited as the SR that had been published previously.

When analyzing the SRs that were published after the protocol was published, it was observed that a total of 237 (95.56%) of the manuscripts analyzed did not result in the publication of the systematic review or meta-analysis associated with that protocol. We proceeded to contact the authors by email, finding that only one of 124 authors who had published these documents responded (the SR was ongoing). Likewise, we found a total of 11 SRs associated with the studied protocols identified, indicating a final publication rate of 4.4%. The median (IQR) time identified between protocol publication and review was one (0.5) year.

**Figure 1:**
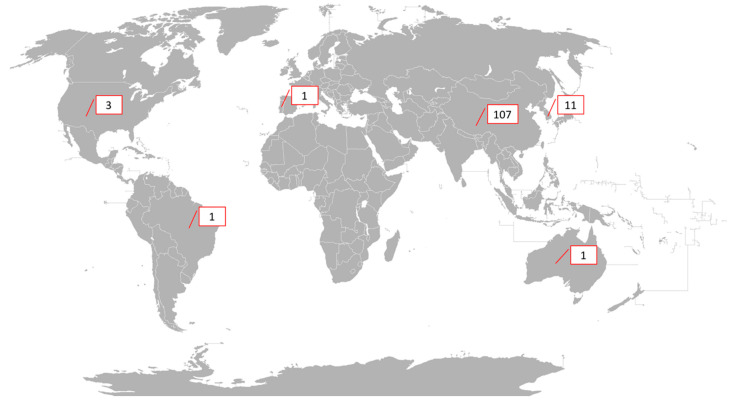
Global distribution of published SRs protocols on acupuncture with quotations. Figure adapted from Fobos92’s world map with CC BY-SA 3.0 license.

By country, determined by the correspondence author’s affiliation, the SR protocols and meta-analyses published were those that received quotation (124 protocols) in: (i) China (107; 86.29%); (ii) South Korea (11; 8.87%); (iii) USA (3; 2.41%); (iv) Australia (1; 0.81%); (v) Brazil (1; 0.81%); and (vii) Portugal (1; 0.81%; Figure 1).

## 4. Discussion

In relation to our primary aim, the correct quoting of references is an essential component of a scientific article. Inaccuracies may perpetuate inaccurate information, make information misleading, and contribute to undermining the basis for future research if poorly interpreted. The incidence of referencing and quoting errors in medical literature is considerable [24]. There are no studies that have previously assessed to what level the quoting of SR protocols in conventional studies is accurate either in CAM research or in other areas of knowledge.

We have observed a high percentage of incorrect quotations in our study, a situation with a complex explanation. Protocols are not exempt from a kind of variant of the phenomenon of “lazy author syndrome” [25], in which the lack of analysis of the full text may lead authors to assume that the abstract of the registry they are consulting through MEDLINE, as an example, may be an SR, a finalized meta-analysis, and not a protocol. It does not help to prevent this process that some journals that publish protocols allow for not stating in the title of the manuscript that it is an SR and meta-analysis protocol [26,27,28], contrary to PRISMA criteria for SR and meta-analysis protocols [29], and more surprisingly not according to their own instructions to authors [30].

Anyway, although the title clearly indicates that what is being read is an SR protocol and meta-analysis [31], and that there is no evidence at this time, it is quoted as a published meta-analysis whose results are favorable to the effect of acupuncture on knee osteoarthritis: “A recent meta-analysis has reported the beneficial effect of acupuncture in the management of knee osteoarthritis” [32]. Even the protocol [33] quotation can be as inaccurate as providing quantitative results: “They found that eight weeks of EA increases CSBMs to 3 or more mean weekly CSBMs in the electro acupuncture group, 37.7% compared with 14.1% in the placebo group” [34]. The problem can be further complicated by inaccurate quotations included in literature reviews; an example is the quotation of this protocol on auricular acupuncture for premature ovarian insufficiency [35]: “Luo et al. (...) conducted a systematic review and meta-analysis of the efficacy and safety of ear acupuncture in the treatment of POI by searching multiple databases before August 2020. The results showed that stimulation of specific ear points can enhance the physiological functions of specific parts of the human body, promote qi and blood circulation, and regulate the reproductive function of the hypothalamus-pituitary-ovarian axis” [36]. This is particularly disturbing given the specific nature of the information derived from a protocol in which the authors state: “We expect to provide reference for premature ovarian insufficiency treatment in the field of traditional Chinese medicine”. This effectively sets a time frame for the literature screenings by August 2020 but never yields any results and certainly none of the quoted data. The present example may be paradigmatic and shows how worrying this problem can be since the erroneous quotation has been incorporated into a review on the CAM treatment of premature ovarian failure. A literature review is one of the highest standards of scientific evidence and it is just a matter of time before this misquotation is reproduced by other authors attending to the literature gathered in the review.

Finally, it also has been observed that in some cases, the protocol [37] is quoted inappropriately: “A recent 2019 overview of systematic reviews and meta-analyses of acupuncture for dysphagia24 suggests that evidence may still be insufficient to produce conclusive evidence, although some symptoms associated with dysphagia may improve” [38]. In this case, the systematic review associated with it was published earlier [39].

Secondly, we found a final publication rate of SRs with respect to the protocols preceding them of 9.1%, which is lower than the recently published rate of 13% [23] in the field of acupuncture research. The results obtained are not comparable because the databases used and the methodology adopted differ significantly. Kim et al. used the MEDLINE and Embase databases up to January 2020, identifying a total of 153 protocols vs. the 250 protocols finally included in our study based on a SCOPUS search. If we analyze the results obtained up to January 2020, the publication rate we have reported in our study would be 14%, which is closer to the rate reported recently by Kim and colleagues. Our results agree with those of Kim et al. in terms of the journals where most protocols are published, with Medicine (77.8% in our study vs. the 71% in that of Kim et al.) first, followed by BMJ Open (15.7% in our study vs. the 21% in that of Kim et al.). Again, the difference in the results observed is probably attributable to the different databases used as well as the time frame considered.

The results shown in our study should make us wonder, first, what is happening regarding the high rate of SR and meta-analysis protocols in acupuncture research that do not apparently conclude in definitive SR and meta-analyses, and, second, what are the reasons for the high misquoting rate and its consequences for the body of knowledge.

The first question has been addressed previously in the literature. The reasons identified that lead (in general) to the non-publication of an SR usually include a lack of time and a rejection of the manuscript by scientific journals [40]. Other more up-to-date reasons are associated with the increased publication of protocols in scientific journals that have weak (or non-existent) peer review processes for those protocols that have been previously registered in public repositories such as PROSPERO [23]. In terms of evaluation of the research, once the protocol is published in a scientific journal, would the publication of the SR and a complete meta-analysis (and the passing of a complete peer review process) have any additional advantages for the researchers? The analysis of published protocols showed that some research groups have published up to four protocols of SR and meta-analysis within the same year and without any evidence that the associated reviews have been conducted. This leads us to what Kim et al. call “the protocol paradox” of SRs [23] since, in the context of the academic institutions of some countries, the publication of the protocol in a scientific journal may be sufficiently relevant for researchers to not end up publishing the final version of the SR.

This study has some limitations, firstly, related to the use of SCOPUS. Although SCOPUS coverage is large enough to identify most of the SR protocols and meta-analyses on acupuncture published in the literature, it may be insufficient to detect the presence of the SRs associated with those protocols and published in journals outside the coverage. Secondly, limitations concern the difficulty to establish contact with the authors to corroborate the possible publication of their SRs or meta-analysis associated with the protocols in other journals not covered by SCOPUS. These situations could affect the determination of the exact nature of the origin of the inaccuracy in the quotes detected.

## 5. Conclusions

Nowadays, SR and meta-analysis are two of the highest standards of scientific evidence. Developing an SR involves covering the full range of scientific methods available to synthesize and classify the information to be included, of which does not meet the criteria for inclusion, to produce higher-quality reviews.

Currently, the number of SR protocols and meta-analyses protocols published in scientific journals and indexed by databases exceeds the publication capacity of the SRs associated with them. These protocols are gradually becoming part of the body of knowledge and the inaccuracy in quoting them contributes to the erroneous quotations attributed to protocols. Due to the speed at which the publication of SR protocols and meta-analyses in scientific journals is growing and considering the fact that, in general, these do not make any novel contribution to knowledge, it is unpredictable how they may affect the knowledge derived from the CAMs.
